# Respiratory Support for Pharmacologically Induced Hypoxia in Neonatal Calves

**DOI:** 10.1155/2016/2129362

**Published:** 2016-02-21

**Authors:** C. G. Donnelly, C. T. Quinn, S. G. Nielsen, S. L. Raidal

**Affiliations:** ^1^Cornell University College of Veterinary Medicine, Ithaca, NY 14850, USA; ^2^School of Animal and Veterinary Sciences, Charles Sturt University, Wagga Wagga, NSW 2650, Australia; ^3^Quantitative Consulting Unit, Research Office, Charles Sturt University, Wagga Wagga, NSW 2650, Australia

## Abstract

Practical methods to provide respiratory support to bovine neonates in a field setting are poorly characterised. This study evaluated the response of healthy neonatal calves with pharmacologically induced respiratory suppression to nasal oxygen insufflation and to continuous positive airway pressure (CPAP) delivered via an off-the-shelf device. Ten calves were randomised to receive either nasal oxygen insufflation (Group 1, *n* = 5) or CPAP (Group 2, *n* = 5) as a first treatment after induction of respiratory depression by intravenous administration of xylazine, fentanyl, and diazepam. Calves received the alternate treatment after 10 minutes of breathing ambient air. Arterial blood gas samples were obtained prior to sedation, following sedation, following the first and second treatment, and after breathing ambient air before and after the second treatment. Oxygen insufflation significantly increased arterial oxygen partial pressure (PaO_2_) but was also associated with significant hypercapnia. When used as the first treatment, CPAP was associated with significantly decreased arterial partial pressure of carbon dioxide but did not increase PaO_2_. These results suggest that the use of CPAP may represent a practical method for correction of hypercapnia associated with inadequate ventilation in a field setting, and further research is required to characterise the use of CPAP with increased inspired oxygen concentrations.

## 1. Introduction

Respiratory dysfunction is recognised as a major source of mortality and morbidity in the bovine neonate [1, 2]. Prematurity, asphyxia, meconium aspiration, infection, and persistent pulmonary hypertension may play a role in the development of respiratory dysfunction and resultant hypoxaemia, hypercapnia, and acidosis [3]. Respiratory compromise can further contribute to an impaired ability to maintain homeostasis and thermogenesis, thereby reducing calf vitality [3, 4].

Respiratory disease in the bovine neonate has been reviewed previously [3, 5]. Conditions resulting from maladaption to the extrauterine environment are more likely to be seen in preterm neonates [6] and are primarily manifest as respiratory distress syndrome (RDS). The primary cause of RDS is a lack of surfactant; however, meconium aspiration and vascular shunting may contribute to respiratory dysfunction. The incidence of this syndrome has been estimated to be as high as 66% in calves, with one-third of farms reporting at least one loss to the condition each year [7]. Calves with increased muscling and calves born after Caesarian section are at greater risk for development of RDS; hence the incidence of the condition may be higher in neonates of increased genetic or financial value [7–10].

There is little information available in the literature on respiratory support of neonatal cattle, as well as an overall deficiency of evidence for respiratory support techniques in large animal neonatology other than in foals [11]. Treatment of respiratory dysfunction in the bovine neonate is poorly documented and seldom practiced due to the lack of supervision at birth and the limited availability of equipment and expertise to treat bovine neonates. The application of intranasal oxygen insufflation, although not commonly used, has been reported for bovine neonates with respiratory compromise [3, 12–14]. However, there is evidence of detrimental effects of oxygen delivery on respiratory physiology and cerebral blood flow [15, 16] and treatment failure may occur due to vascular shunting [13], hypoventilation, prematurity, and decreased surfactant [14]. Synchronised intermittent mandatory ventilation has been used to mechanically ventilate calves that did not respond to oxygen insufflation [13, 17] in a dedicated research and specialist hospital facility, with modest survival to discharge. Pharmacological agents, such as doxapram and methylxanthines, have been used as respiratory stimulants [18] but have the potential to reduce cerebral blood flow and may result in negative long-term neurological complications.

Continuous positive airway pressure (CPAP) has been advocated in human neonatology and cardiopulmonary resuscitation. The use of the technique for ventilatory support of human patients was reported as early as the 1930s [19, 20], but use in neonates was not described until much later [21]. In combination with the administration of exogenous surfactant and preterm glucocorticoid administration, CPAP is now widely used for respiratory support of human neonates [22–24]. CPAP primarily improves ventilation but may also have a role in improving intrapulmonary perfusion by increasing transpulmonary pressure (as inspiratory and expiratory pressure are maintained higher than ambient pressure), resulting in an increased functional reserve capacity (FRC). Increased FRC has several follow-on effects including reduced work of breathing, redistribution of lung fluid, reduction in airway resistance, decreased intrapulmonary shunting, and improved surfactant function. Cumulatively these responses improve oxygenation and reduce the partial pressure of carbon dioxide. This is advantageous for neonatal respiratory support as these patients are subject to reduced FRC, atelectasis, and increased work of breathing. The technique is technically less demanding than true ventilatory techniques and is less invasive than mechanical ventilation. It is therefore more readily available and is not associated with complications such as bronchopulmonary dysplasia or tracheal necrosis.

To date there has been limited evaluation of CPAP for respiratory support of neonates in the veterinary literature. Use of the technique in preterm neonatal lambs resulted in a greater increase in lung compliance, volume, and FRC than mechanical ventilation or no treatment [6]. More broadly, Briganti et al. [25] demonstrated increased arterial oxygen partial pressure following CPAP treatment of sedated adult dogs, compared to oxygen insufflation alone. The technique has been used in anaesthetised adult horses [26–28].

The rise in advanced reproduction techniques including* in vitro* embryo production and cloning, combined with the increased economic value of these calves, suggests that the incidence and inclination to intervene in bovine neonatal respiratory disease will increase [13], and this warrants the development of appropriate therapies for respiratory support of newborn calves [29]. Investigations into improved methods of support must include scope for ease of treatment and cost of equipment and utility in a field setting. As CPAP is less demanding than ventilation, CPAP may present an appropriate technique for use in the bovine neonate. The adaptation of techniques utilising readily available portable compressor driven devices is particularly attractive as a field treatment for neonatal calves without the need for supplemental oxygen, advanced expertise, or more invasive ventilator support. The present study was undertaken as a pilot study to evaluate the effect of oxygen insufflation and a portable off-the-shelf CPAP device on arterial blood gas measurements in calves delivered by Caesarean section and following pharmacological induction of hypoxaemia.

## 2. Materials and Methods

### 2.1. Experimental Animals

Six male (BW 38 ± 7 kg) and six female (BW 37 ± 4.4 kg) Angus (*n* = 10) and Angus-Hereford cross (*n* = 2) calves were made available for inclusion in this study following elective Caesarean delivery (gestation day 275 ± 7). Surgeries occurred over two days with six calves delivered on each day. Cows were pretreated on gestation day 274 ± 7 with dexamethasone (0.44 mg/kg, Dexafort 3 mg/mL, Intervet, East Bendigo, Australia) and received preoperative procaine penicillin (Depocillin 300 mg/mL, MSD Animal Health, East Bendigo, Australia) (40 mg/kg by intramuscular injection) and clenbuterol (Planipart 30 *μ*g/mL, Boehringer Ingelheim, North Ryde, Australia) (0.65 mg/kg by intramuscular injection). At delivery calves were suspended vertically by their hind legs for a period of 30 seconds. Neonatal viability was assessed at this time and again after five minutes using a modified Apgar score adapted from Born [30]. Calves were allowed to nurse from their dam, with time to stand and nurse recorded. Calves were considered suitable for inclusion in this experiment if they had an Apgar score ≥6 at five minutes postpartum and physical examination within normal limits at 20–24 hours of age. All procedures were undertaken at 20–24 hours postpartum and were approved by the Animal Care and Ethics Committee at Charles Sturt University (ACEC 12/037).

### 2.2. Experimental Method

Calves were restrained in left lateral recumbency on foam matting for the duration of the experiment. Calves were not sedated before restraint nor during preparation for sampling. The right jugular grove and left medial antebrachium were clipped and surgically prepared with chlorhexidine and alcohol. A 16-gauge two-and-a-half-inch catheter (Surflo catheter, Terumo, Tokyo, Japan) was placed in the right jugular vein and a 22-gauge one-inch catheter (Surflo catheter, Terumo, Tokyo, Japan) was placed in the left brachial artery. Catheters were secured with polyacrylamide adhesive and patency was maintained with heparinised saline (5 IU/L). Data collection began immediately following placement of these catheters (T0) with collection of both venous and arterial samples. Venous samples were collected into EDTA and serum vacutainers (BD Australia, North Ryde, Australia) for complete blood count and serum biochemistry. Arterial samples were collected anaerobically into preheparinised syringes (BD Preset Arterial Blood Gas Syringe, BD Australia, North Ryde, Australia) for blood gas analysis, as described below.

Respiratory depression was induced by treatment with diazepam (Pamlin 5 mg/mL, Parnell, Alexandria, Australia) (0.1 mg/kg IV), xylazine hydrochloride (Xylazil 10 mg/mL, Troy Laboratories, Glendenning, Australia) (0.01 mg/kg IV), and fentanyl (fentanyl 50 *μ*g/mL, DBL Hospira, Melbourne, Australia) (3 *μ*g/kg IV). At this time and throughout the course of the experiment calves were monitored with an oxygen saturation probe (Masimo Radical, Masimo Australia, Frenchs Forest, Australia). Arterial samples were collected anaerobically as follows:

 T0: baseline—before tranquilization with calves breathing ambient air.
 T1: ten minutes after administration of sedation, calves breathing ambient air.
 T2: ten minutes after initiation of respiratory support (CPAP or oxygen insufflation).
 T3: rest—ten minutes following discontinuation of initial respiratory support, breathing ambient air.
 T4: ten minutes after initiation of alternate respiratory support (oxygen insufflation or CPAP).
 T5: recovery—ten minutes following discontinuation of second respiratory intervention, breathing ambient air.

Heart rate, rectal temperature, and oxygen saturation data were also collected at each time point. Oxygen saturation was determined by pulse oximetry (Masimo Radical, Masimo Australia, Frenchs Forest, Australia), with the probe attached to the lip (nonpigmented animals) or tongue. At the conclusion of the experiment calves were administered with an equipotent dose of atipamezole (Antisedan 5 mg/mL, Pfizer Animal Health, West Ryde, Australia) (0.01 mg/kg IM) to reverse the effects of xylazine.

### 2.3. Respiratory Support

Both CPAP and oxygen insufflation were delivered via a standard canine anaesthesia mask following validation of mask fit and pressure maintenance using a pressure manometer during CPAP in pilot cadaver studies. Mask dead space was approximately 250 millilitres, measured by water displacement. The mask used to provide CPAP was modified to accommodate exhaust gases by drilling a series of holes into the upper portion of the mask to allow for the escape of gas during expiration and thereby prevent mask seal failure. CPAP was provided using a titrated off-the-shelf constant pressure generator (Resmed 8, ResMed Inc., Bella Vista, Australia). The machine generated a positive pressure via compressor, delivering room air at 10 cm H_2_O. Air delivered via the CPAP apparatus was not humidified and did not include a ramp. Humidified oxygen was delivered through standard oxygen tubing to the mask at a rate of 5 L/min.

### 2.4. Blood Gas Analysis

Samples obtained anaerobically from the brachial artery catheter into preheparinised syringes were immediately placed on ice and processed within 4 hours of collection [31]. Samples were processed in order of collection. Arterial blood pH, partial pressure of oxygen (PaO_2_), partial pressure of carbon dioxide (PaCO_2_), lactate, bicarbonate (HCO_3_^−^), and oxygen saturation were determined using a bench-top analyser (GEM Premier 3500, Instrumentation Laboratory, Brisbane, Australia), with values adjusted for contemporaneous rectal temperature.

### 2.5. Experimental Design and Statistical Analysis

A randomised block design was utilised with first treatment (oxygen insufflation or CPAP) assigned by random number generation. Pairs of calves were considered one replicate, with each calf of a replicate pair receiving a different first treatment. For analysis, Group 1 calves received oxygen supplementation (5 L/min) at T2, followed by CPAP at T4; Group 2 calves received CPAP at T2 and oxygen insufflation at T4. Analysis of covariance (ANCOVA) was performed using the statistical software R (GNU Operating Systems, MIT, Cambridge, USA) with time and treatment (oxygen supplementation or CPAP) as fixed effects and calf and replicate as covariates. Data distribution was tested for normality using Levine's test. Weighted analysis was performed on nonnormal data.* Post hoc* evaluation was performed using Tukey's test to verify independence, with differences considered significant for *P* ≤ 0.05. Respiratory support (oxygen insufflation and CPAP) and treatment order were further evaluated by two-way repeated measures analysis of variance with time (T2 and T4) and treatment (O_2_ and CPAP) as factors. Unless otherwise stated, data are presented as mean ± standard deviation.

## 3. Results

### 3.1. Clinical Observations

Twelve calves were available for inclusion in the experiment. One calf was excluded at the beginning of the experiment because a patent arterial cannulation could not be established. A second calf was excluded due to an umbilical infection, detected at the preexperiment physical exam. Data is presented from the remaining five male (BW 39.7 ± 7 kg) and five female calves (38.1 ± 3.7 kg). Birth weight (39.4 ± 6.6) for Group 1 calves (receiving oxygen at T2 and CPAP at T4) was not significantly different to that recorded for Group 2 calves (35.6 ± 4.8) (*P* = 0.11). The Apgar score was greater at five minutes postpartum (7.5 ± 0.7) than immediately after birth (5.6 ± 1.2), but this difference was not significant (*P* ≥ 0.05). All calves met the inclusion criterion of an Apgar score ≥6 at five minutes postpartum. Mean score for Group 1 calves (4.8 ± 0.8) was significantly less than that of Group 2 calves (6.6 ± 0.9) immediately after birth (*P* = 0.01), but differences were not significant by 5 minutes postpartum (7.8 ± 0.4, Group 1; 7.0 ± 0.7, Group 2; *P* = 0.07).

Both mask insufflation and mask delivered CPAP were well tolerated. No adverse response to the placement of the mask, or to the sound of the machine, was observed during this experiment and no attempt was made by calves to remove the mask. Mask seal failure during CPAP, recognised as machine alerts, was observed infrequently, and all failures were immediately rectified by adjustment of the mask position. Subjectively, the expiratory effort of calves was mildly increased during CPAP treatment when compared to efforts observed during oxygen insufflation or between treatments whilst breathing ambient air. No complications attributable to treatment with CPAP or oxygen were encountered during the experiment.

**Figure 1:**
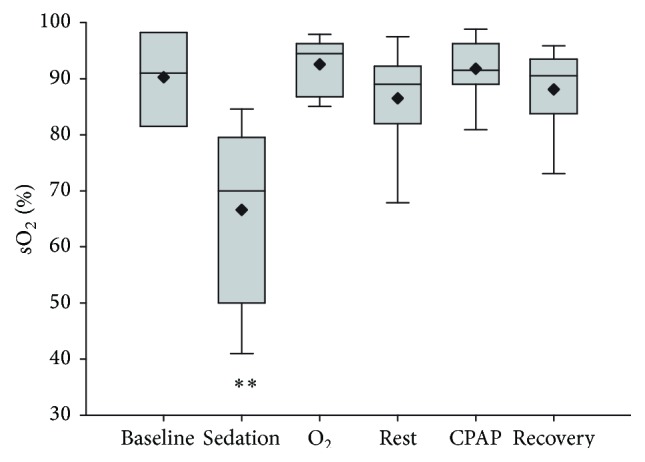
Haemaglobin saturation determined by pulse oximetry following sedation and respiratory support by oxygen insufflation or continuous positive airway pressure (CPAP). Values are shown as median (horizontal line), mean (diamond), quartiles, and range immediately after catheter placement (T0, baseline), 10 minutes following sedation (T1, sedation), following 10 minutes of oxygen supplementation (O_2_), following 10 minutes of CPAP, and after 10 minutes of breathing ambient air following respiratory support (T3, rest, and T5, recovery). Order of respiratory support (O_2_ supplementation or CPAP) was randomly assigned to replicate groups of paired calves and administered at T2 (5 calves) or T4 (5 calves); treatment order had no significant effect on response to O_2_ supplementation or CPAP. Values obtained following sedation were significantly less than at all other times (^*∗∗*^ *P* < 0.001).

Calves were markedly affected by the sedation protocol, which consistently produced recumbency and obtunded mentation. Oxygen saturation (sO_2_) was significantly reduced following sedation (*P* < 0.01, Figure 1) and was increased following respiratory support. Treatment order (CPAP or O_2_ insufflation as first treatment) did not have a significant effect on the observed response. Recovery following reversal was variable between calves, with most regaining ambulation within four hours. Two calves did not regain ambulation for more than four hours, with one of these calves treated with intravenous fluids and naloxone. Both calves were able to maintain sternal recumbency following reversal, and by 24 hours after the experiment all calves demonstrated no residual effects. At one week of age calves included in the experiment had gained equivalent amounts of weight to calves that were delivered at the same time and not included in the experiment.

### 3.2. Blood Gas Analysis

**Figure 2:**
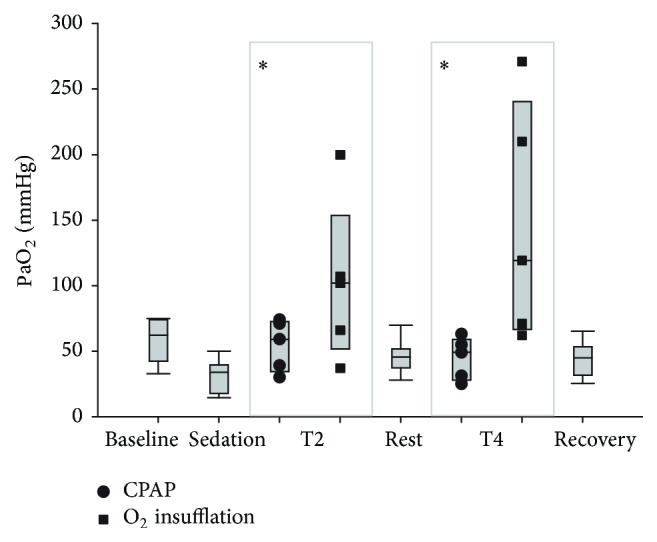
Arterial partial pressure of oxygen (PaO_2_) following respiratory support by oxygen insufflation or continuous positive airway pressure (CPAP). Values are shown as median (horizontal line) and quartiles and range immediately after catheter placement (T0, baseline), 10 minutes following sedation (T1, sedation), following 10 minutes of oxygen supplementation (O_2_), following 10 minutes of CPAP, and after 10 minutes of breathing ambient air following respiratory support (T3, rest, and T5, recovery). Individual results following respiratory support (CPAP or O_2_ insufflation) are shown at T2 and T4. Order of respiratory support at T2 (CPAP or O_2_ insufflation) was randomly assigned to replicate groups of paired calves. The alternate treatment (O_2_ insufflation or CPAP) was administered at T4. Values obtained following O_2_ insufflation were significantly greater than values obtained following sedation (*P* < 0.05). Treatment order had no significant effect on response to O_2_ supplementation or CPAP.

Time of sampling had a significant effect (*P* = 0.001) on PaO_2_, and there was a significant time and treatment interaction (*P* = 0.000). Sedation decreased PaO_2_ values from 57.8 ± 16.9 mmHg at T0 to 31.5 ± 11.9 mmHg, although this difference was not significant (Figure 2). Oxygen insufflation, but not CPAP, caused increased PaO_2_ relative to postsedation values. This effect was significant at both T2 and T4 and although values obtained at T2 (102.4 ± 61.5 mmHg) were less than those obtained at T4 (146.6 ± 91.0 mmHg), this difference was not significantly different. There was no apparent effect on PaO_2_ attributable to CPAP at T2 (54.6 ± 19.4 mmHg) or T4 (44.6 ± 16.1 mmHg), although values obtained were greater than was observed following sedation (31.5 ± 11.9 mmHg).

**Figure 3:**
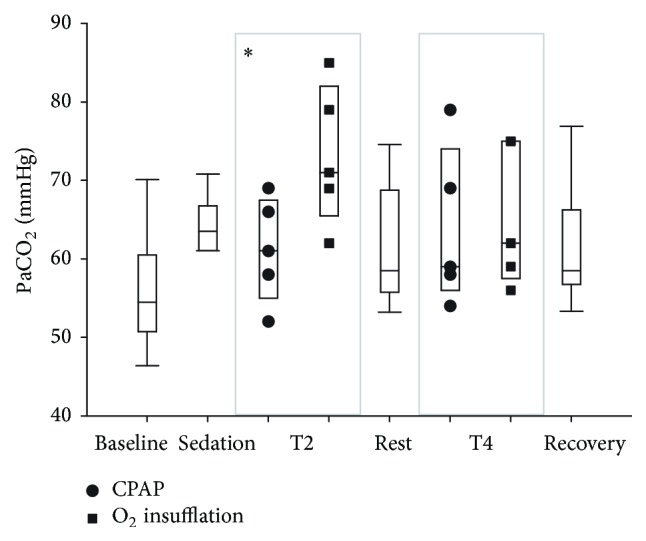
Arterial partial pressure of carbon dioxide (PaCO_2_) following respiratory support by oxygen insufflation or continuous positive airway pressure (CPAP). Values are shown as median (horizontal line) and quartiles and range immediately after catheter placement (T0, baseline), 10 minutes following sedation (T1, sedation), following 10 minutes of oxygen supplementation (O_2_), following 10 minutes of CPAP, and after 10 minutes of breathing ambient air following respiratory support (T3, rest, and T5, recovery). Individual results following respiratory support (CPAP or O_2_ insufflation) are shown at T2 and T4. Order of respiratory support at T2 (CPAP or O_2_ insufflation) was randomly assigned to replicate groups of paired calves. The alternate treatment (O_2_ insufflation or CPAP) was administered at T4. CPAP was associated with significantly lower PaCO_2_ values than was evident following O_2_ insufflation at T2 (^*∗*^ *P* < 0.05).

Significant time (*P* = 0.009) and treatment (*P* = 0.012) effects were observed on arterial PaCO_2_, although treatment and time interactions were not significant. Arterial CO_2_ partial pressure was increased following sedation (64.5 ± 3.5 mmHg, mean ± sd) compared with baseline values (55.7 ± 7.2 mmHg), and oxygen insufflation was associated with a further increase in PaCO_2_ (69.3 ± 9.4 mmHg, Figure 3). This trend was more marked in calves receiving oxygen insufflation at T2, 10 minutes following sedation. When order of treatment was taken into account, CPAP resulted in significantly lower PaCO_2_ levels if it was used as a first treatment (T2) than was observed following oxygen supplementation at this time (*P* < 0.05).

**Figure 4:**
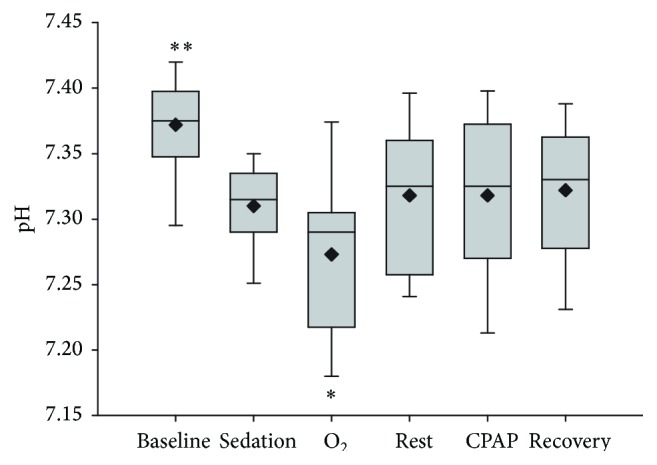
Arterial blood pH following sedation and respiratory support by oxygen insufflation or continuous positive airway pressure (CPAP). Values are shown as median (horizontal line) and mean (diamond) and quartiles and range immediately after catheter placement (T0, baseline), 10 minutes following sedation (T1, sedation), following 10 minutes of oxygen supplementation (O_2_), following 10 minutes of CPAP, and after 10 minutes of breathing ambient air following respiratory support (T3, rest, and T5, recovery). Order of respiratory support (O_2_ supplementation or CPAP) was randomly assigned to replicate groups of paired calves and administered at T2 (5 calves) or T4 (5 calves); treatment order had no significant effect on response to O_2_ supplementation or CPAP. Baseline results were significantly higher than results obtained at all other times (^*∗∗*^ *P* < 0.001), and results obtained following O_2_ supplementation were significantly lower than all other results (^*∗*^ *P* < 0.005).

Blood pH was significantly affected by treatment (*P* = 0.050) and time (*P* = 0.007). Consistent with changes in PaCO_2_, pH was decreased following sedation (7.31 ± 0.03) and after oxygen treatment (7.27 ± 0.06), when compared to baseline results (7.37 ± 0.04) (Figure 4). There was no significant interaction between treatment and time, and order of treatment had no effect on plasma pH (*P* > 0.05).

**Figure 5:**
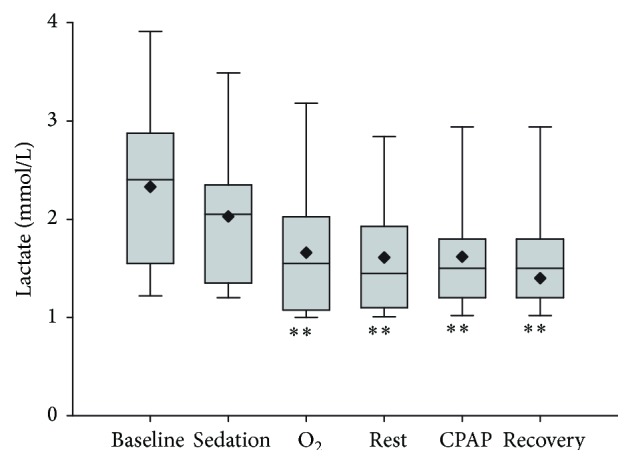
Arterial blood lactate concentration following respiratory support by oxygen insufflation or continuous positive airway pressure (CPAP). Values are shown as median (horizontal line) and mean (diamond) and quartiles and range immediately after catheter placement (T0, baseline), 10 minutes following sedation (T1, sedation), following 10 minutes of oxygen supplementation (O_2_), following 10 minutes of CPAP, and after 10 minutes of breathing ambient air following respiratory support (T3, rest, and T5, recovery). A significant time effect was observed, as results obtained after sedation were significantly less than baseline (^*∗∗*^ *P* < 0.001). Order of respiratory support (O_2_ supplementation or CPAP) was randomly assigned and had no significant effect on response to O_2_ supplementation or CPAP.

A significant time (*P* = 0.001) effect was observed on blood lactate concentrations (Figure 5). Lactate concentrations were significantly (*P* < 0.001) lower in all samples obtained after sedation than in the baseline sample. Order of treatment did not have a significant effect on measured lactate concentrations and an interaction between treatment and time was not detected. Neither oxygen supplementation nor CPAP affected (*P* > 0.05) arterial blood bicarbonate concentrations, and there was no significant effect of time or time-treatment interaction detected for this analyte (data not shown).

## 4. Discussion

Consistent with previous reports [12], the current study demonstrated that oxygen insufflation effectively increased PaO_2_ in hypoxaemic calves. All calves in the current study showed significantly higher PaO_2_ levels immediately after treatment with oxygen insufflation delivered through a mask. Other studies have suggested that the positive response to oxygen insufflation may not be apparent in all treated calves, particularly those with incomplete transition from foetal circulation and resultant left to right shunting [13]. Further, in neonates with RDS, especially preterm neonates, atelectasis due to a relative or absolute deficiency in surfactant may compromise the ability to ventilate and hence limit response to oxygen supplementation [14]. Calves in the current study were born at term or close to term and had antenatal exposure to exogenous glucocorticoids. It is unlikely, therefore, that an absolute or relative deficiency of surfactant had a significant impact on respiratory measures in this study. Arterial blood gases following oxygen insufflation in many calves in the current study were well above physiological requirements. Supraphysiologic oxygen partial pressure may potentiate oxidative stress and be deleterious in neonates [15, 32] due to reflex vasoconstriction of arteriolar smooth muscle and consequent reduced blood flow to the brain, heart, and kidney [16].

Minor increases in PaO_2_ were observed in most calves following treatment with CPAP, although observed differences were not significant. Power analysis of data from this study suggested that inclusion of an increased number of animals (*n* = 7) would demonstrate a significant treatment effect (*α* = 0.05, 0.80) if these findings are reproducible. As CPAP provides constant pressure throughout the respiratory cycle it prevents the collapse of small airways and alveoli, increasing the FRC. Intrinsically CPAP maintains and to an extent increases the surface area of exchange barriers, potentially allowing increased PaO_2_ by reducing ventilation deficits rather than by increasing the oxygen gradient. Thus the increased PaO_2_ observed in the current study associated with CPAP delivered room air, whilst small, may indicate the prevention of atelectasis.

Oxygen insufflation was associated with a significant increase in PaCO_2_ in the current study, and this effect was more pronounced when oxygen was used as a first treatment. Although expired gas measurements were not taken in the current experiment, it was speculated that hypercapnia resulted from a reduced respiratory drive with a consequent decrease in minute ventilation and resultant increased alveolar partial pressure of CO_2_. Alternatively (or additionally), the administration of supplementary oxygen may increase metabolic rate and hence carbon dioxide production [33].

In contrast to findings subsequent to oxygen supplementation, PaCO_2_ was decreased following CPAP treatment, relative to values obtained following sedation or oxygen insufflation, and this effect was significant when CPAP was used as the first treatment. Hypercapnia has a direct effect on acid-base balance. In the current study, the induction of respiratory depression and treatment with oxygen insufflation resulted in significantly decreased pH, and this effect was prevented or corrected by CPAP. As CPAP prevents alveolar collapse during expiration, the ability for carbon dioxide to diffuse across the pulmonary membrane is increased. The major benefit of CPAP may therefore derive from improved CO_2_ exchange without more invasive ventilatory support.

The experimental model used in the current study was designed to replicate the physiological consequences of respiratory distress via a reversible model of respiratory compromise with limited welfare cost to experimental subjects. However, care must be taken in extrapolating the effects observed in these, otherwise healthy, calves, to individuals with inadequate surfactant production or other respiratory pathology. The respiratory depressant effects of fentanyl and cardiac depressant effect of xylazine are well known in other veterinary species [34–36] but have not been reported in the bovine neonate. The increased PaCO_2_ measurements observed following administration of these agents may be due to synergistic negative effects on respiratory function. Hypoxia and hypercapnia associated with xylazine sedation are primarily from a peripherally mediated increase in the shunt fraction (*Q*_*s*_/*Q*_*t*_) and not from hypoventilation or postural changes [36]. In contrast fentanyl produces direct depression of respiration through a centrally mediated reduction in inspiratory effort and frequency. In humans the effect of fentanyl is dose limiting; however, in animal species examined it is dose dependent and plateaus and is considered mild [35].

Lateral recumbency may affect blood gas pressure [37, 38]. Calves included in this study were slightly hypercapnic at inclusion, relative to previously published values for calves of this age [12, 13]. In light of the relatively normal PaO_2_ levels at this time, the higher PaCO_2_ results were attributed to a mild left to right shunt or possibly due to positioning in lateral recumbency. Hence, it is possible that calves in the current study had a degree of respiratory dysfunction beyond that induced pharmacologically. However inclusion criteria ensured that they were not affected by overt primary respiratory dysfunction and, as such, the largest effect on the experimental respiratory depression and observed PaO_2_ and PaCO_2_ was the administration of pharmacologic agents. This implies that PaO_2_ and PaCO_2_ would likely improve as the pharmacologic agents were eliminated, a consideration supported by the stronger treatment effects observed at T2 than T4. In the current study, such considerations were controlled for by inclusion of alternate pairs of calves (rather than individuals) as the experimental unit, as well as by randomisation of the first treatment. This ensured that bias was not introduced by additive treatment effects or from the waning action of pharmacological agents employed.

The results of the present study demonstrate that whilst oxygen supplementation alone may address hypoxia, this technique may be insufficient for calves with inadequate surfactant and/or hypoventilation. Our results suggest that CPAP may address hypercapnia and therefore be a useful adjunctive treatment for the management of respiratory distress in bovine neonates. Calves used in the current study were a convenience sample of healthy animals, and further studies with larger populations and evaluating spontaneous disease are warranted.

The CPAP methodology used in the current study shows promise as a field treatment for respiratory depression in calves as it maintained or improved PaO_2_ at levels comparable with untreated calves and was not associated with hypercapnia. However, in order to optimise the treatment, investigations into CPAP with an increased inspired oxygen content/concentration (or partial pressure) are warranted. An off-the-shelf at home compressor driven device was evaluated as a simple, readily available, noninvasive, and inexpensive method of respiratory support. Whilst this form of CPAP did not cause harm to the animals, some inadequacies were identified. Increased expiratory effort was observed, likely due to the generation of a constant airflow, with titrated inspiratory and expiratory pressure. In calves, the initial phase of expiration is passive recoil, followed by an active component. An increase in the active component of respiration may lead to fatigue of the muscles involved in respiration [32]. To avoid this, human neonatal systems generally use fluidic flow devices to produce the distending pressure. In addition they also have an expiratory limb similar to that used in a circle anaesthesia circuits. This results in the flow reversing by the Coanda effect during expiration, whilst maintaining airway pressure, and therefore does not lead to an exaggerated expiratory effort [32] and would negate the need for venting of expired gases through the mask.

## 5. Conclusion

The institution of mask delivered CPAP was well tolerated by subjects and did not result in adverse outcomes in the current experiment. Improvements in blood gas parameters were modest and suggested that CPAP may be of primary benefit in preserving eucapnia. Whilst the use of oxygen insufflation in hypoxaemic calves effectively increased arterial oxygen tensions to supraphysiologic levels, this treatment was associated with increased arterial carbon dioxide concentrations and decreased pH, presumably due to reduced respiratory drive. The pharmacologically induced hypoxaemia, hypercapnia, and acidaemia in this experiment model the physiological consequences of respiratory insufficiency well and, as such, provide a platform for further investigations aimed at optimising CPAP delivery. However, the pharmacological induction of respiratory compromise does not replicate spontaneous disease, and the results of the current study justify further investigation into the use of CPAP in naturally occurring disease.
